# Immunogenic Cell Death in Melanoma Induced by Radiotherapy, Photodynamic Therapy, or Non-Thermal Plasma: The Role of Reactive Oxygen and Nitrogen Species

**DOI:** 10.3390/biomedicines14092135

**Published:** 2026-09-21

**Authors:** Lisa Van der heyden, Steve Vanlanduit, Evelien Smits, Annemie Bogaerts, Angela Privat-Maldonado

**Affiliations:** 1 Research Group PLASMANT and Center of Excellence PLASMA, University of Antwerp, 2020 Antwerp, Belgium; annemie.bogaerts@uantwerpen.be (A.B.); angela.privatmaldonado@uantwerpen.be (A.P.-M.); 2 Center for Oncological Research, University of Antwerp, 2610 Antwerp, Belgium; evelien.smits@uza.be; 3 Industrial Vision Lab, University of Antwerp, 2020 Antwerp, Belgium; steve.vanlanduit@uantwerpen.be

**Keywords:** melanoma, reactive oxygen and nitrogen species (RONS), immunogenic cell death (ICD), radiotherapy, photodynamic therapy, non-thermal plasma (NTP)

## Abstract

Melanoma is characterised by profound immune evasion, and although immune checkpoint inhibitors (ICIs) have transformed clinical management, durable responses remain limited to a subset of patients. Therapies that induce immunogenic cell death (ICD) can enhance anti-tumour immunity by converting dying tumour cells into a source of tumour antigens and danger signals. Radiotherapy (RT), photodynamic therapy (PDT), and non-thermal plasma (NTP) are three pro-oxidant cancer therapies that induce ICD through the generation of reactive oxygen and nitrogen species (RONS), yet these modalities are rarely compared from the perspective of the characteristics of the RONS they produce. This review compares these modalities through a mechanistic framework in which RONS composition, site of generation, lifetime, and diffusion shape the intracellular compartments exposed to oxidative stress and the resulting immune-relevant responses. These characteristics influence which intracellular compartments sustain oxidative damage, thereby shaping endoplasmic reticulum (ER) stress, DNA-damage sensing, and mitochondrial dysfunction. The resulting stress responses can promote damage-associated molecular pattern emission and create conditions that support anti-tumour immune activation. Across the available literature, RT is commonly associated with cytosolic DNA sensing and type I interferon signalling, PDT frequently involves photosensitiser-localisation-dependent ER stress and calreticulin exposure, and NTP can engage several stress pathways in a treatment-parameter-dependent manner. These patterns are overlapping rather than exclusive and depend on the experimental variables of each treatment modality and the tumour biological context. By integrating these mechanistic differences, this review aims to provide a framework for understanding how RONS characteristics shape ICD and its associated anti-tumour immune effects, and how these pro-oxidant therapies may be combined with ICIs in melanoma. Because direct head-to-head comparisons using matched melanoma models and common immunological endpoints remain limited, the proposed modality-associated patterns should be interpreted as context-dependent tendencies rather than a fixed mechanistic hierarchy.

## 1. Introduction

Melanoma remains one of the most aggressive forms of skin cancer because of its high metastatic potential and remarkable capacity for immune evasion [1]. Although immune checkpoint inhibitors (ICIs) targeting PD-1/PD-L1 and CTLA-4 have substantially improved clinical outcomes, durable responses are limited to a subset of patients owing to primary and acquired resistance and immune-related toxicity [2,3]. A major challenge is that many melanomas fail to generate effective tumour-specific T-cell responses or actively suppress anti-tumour immunity, thereby restricting the efficacy of ICI [4,5].

Radiotherapy (RT), photodynamic therapy (PDT), and non-thermal plasma therapy (NTP) are all pro-oxidant cancer therapies that can generate reactive oxygen and nitrogen species (RONS), although they do so through distinct physical and chemical mechanisms [6,7,8]. By disrupting cellular redox homeostasis, therapy-induced RONS can activate stress-response pathways and promote the exposure or release of damage-associated molecular patterns (DAMPs) [9]. These processes can contribute to immunogenic cell death (ICD), a regulated form of cell death that transforms dying tumour cells into a source of tumour antigens and immunostimulatory danger signals [10,11,12]. These shared immunological effects provide a mechanistic rationale for evaluating combinations of pro-oxidant therapies with ICIs [13]. RT, PDT, and NTP can each induce ICD-associated signalling, but differences in RONS composition, site of generation, lifetime, and diffusion may shape the relative involvement of compartment-specific stress pathways [6,9,13,14,15]. These characteristics are expected to influence the relative involvement of ER stress, mitochondrial dysfunction, membrane damage, and DNA-damage sensing, as well as the resulting DAMP profile and immune response. However, the relevant pathways overlap across modalities and are strongly influenced by experimental variables, including radiation dose and fractionation, photosensitiser identity and subcellular localisation, oxygen availability, plasma source and delivery conditions, and the biological properties of the tumour model.

This review examines how differences in the RONS characteristics generated by each therapy shape the induction of ICD and its associated anti-tumour immune effects. Rather than considering RT, PDT, and NTP in isolation, we compare these modalities within a unified framework that links RONS characteristics to compartment-specific cellular stress responses and subsequent DAMP emission. While each modality has been identified as an inducer of ICD, they are rarely compared from the perspective of the RONS characteristics they generate and how these influence their immunogenic effects [6,14,15]. By characterising the mechanisms these therapies share and those that set them apart, this review aims to clarify how RONS characteristics influence immunogenic outcomes and to translate this understanding into a rational basis for combining RT, PDT, and NTP with ICIs in melanoma. Importantly, this comparison represents an integrative synthesis of the available literature rather than a direct head-to-head evaluation, as RT, PDT, and NTP have rarely been investigated using matched experimental models, treatment conditions, and immunological endpoints. The framework proposed here should, therefore, be interpreted as a mechanistically informed model that identifies recurring modality-associated patterns and generates hypotheses for future comparative validation. Melanoma provides a particularly relevant setting for this comparison, as ICI already forms a cornerstone of clinical management, while many cutaneous and superficial lesions remain readily accessible to these local therapies capable of inducing ICD [16,17].

## 2. Therapy-Specific RONS Profiles

### 2.1. Radiotherapy

**Figure 1:**
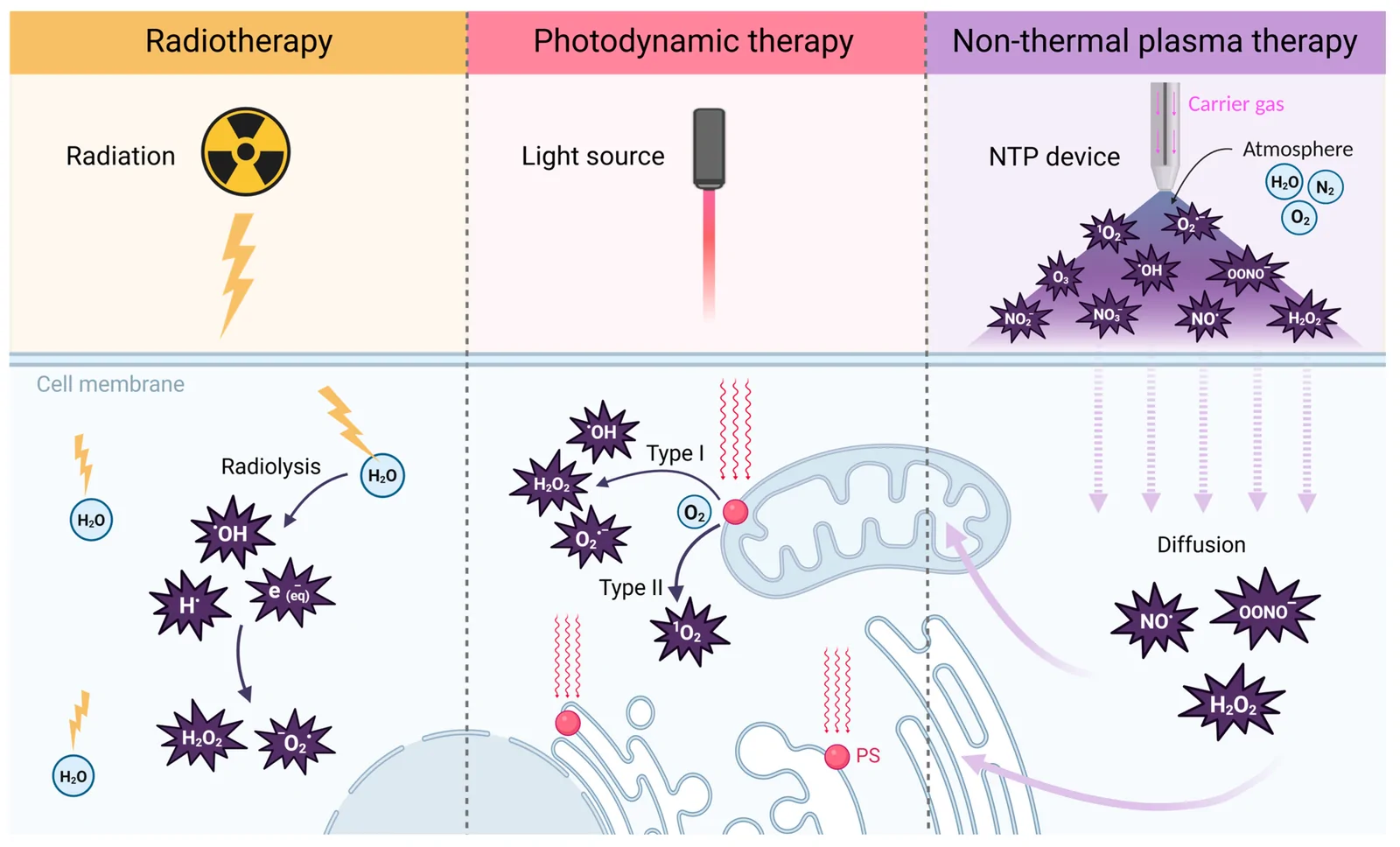
RONS generation by radiotherapy (RT), photodynamic therapy (PDT), and non-thermal plasma therapy (NTP) in tumour cells. Schematic representation of reactive oxygen and nitrogen species (RONS) generation by RT, PDT, and NTP in and around a tumour cell. Each panel highlights the predominant intracellular sites of RONS generation and oxidative stress associated with that modality. In RT (**left**), ionising radiation interacts with intracellular water, leading to radiolysis and the formation of short-lived species, such as •OH, H•, and e^−^(aq), which subsequently generates longer-lived reactive oxygen species (ROS), including H_2_O_2_ and O_2_•^−^. In PDT (**middle**), a photosensitiser (PS), most commonly localised to the endoplasmic reticulum (ER) and mitochondria, is excited by light and, in the presence of O_2_, generates O_2_•^−^, H_2_O_2_, and •OH via type I reactions and ^1^O_2_ via type II reactions. In NTP (**right**), a plasma source produces a rich mixture of both short- and long-lived RONS in the gas phase, among which certain reactive species (H_2_O_2_, NO•, and ONOO^−^) are able to diffuse into the intracellular environment. Created in BioRender. Privat Maldonado, A. (2026): https://BioRender.com/e41zzik.

RT exerts its biological effects by depositing ionising energy within biological tissue (Figure 1, left) [18]. Because water is the principal constituent of cells, ionising radiation induces oxidative stress predominantly through water radiolysis, initiating a cascade of reactions that generate highly reactive oxygen species (ROS), predominantly the hydroxyl radical (•OH), together with hydrated electrons (e^−^_(aq)_) and hydrogen radicals (H•) [19,20]. The hydroxyl radical is the principal oxidising species and the main mediator of indirect damage to biomolecules [21]. These short-lived radicals subsequently drive secondary reactions that generate longer-lived oxidants, including hydrogen peroxide (H_2_O_2_) and superoxide (O_2_•^−^), which persist longer than the primary ROS [22].

Because ionising energy is deposited throughout the irradiated cell, RT generates reactive species across multiple intracellular compartments rather than being confined to a single location [19]. A fraction of this energy is deposited directly within the nucleus, producing clustered DNA lesions alongside a more diffuse oxidative environment in the cytoplasm, mitochondria, and ER [23]. The extent and composition of this oxidative environment depend on radiation parameters, including dose, dose rate, and fractionation, as well as tissue characteristics [18]. For example, Meng et al. [24] demonstrated that, at an equivalent total radiation dose, low-dose-rate irradiation induced greater ROS and mitochondrial superoxide accumulation than high-dose-rate irradiation in normal human cells. These changes were accompanied by changes in mitochondrial morphology and membrane potential, indicating that dose rate can modulate mitochondrial oxidative responses to RT [24].

### 2.2. Photodynamic Therapy

PDT generates RONS through a photosensitiser (PS)- and light-dependent mechanism (Figure 1, middle) [25]. Upon illumination at an appropriate wavelength, the PS is excited to a triplet state. In the presence of molecular oxygen, this enables two principal photochemical pathways. Type I reactions involve electron or hydrogen transfer to surrounding substrates, whereas type II reactions involve direct energy transfer to ground-state oxygen [26]. Type I reactions generate ROS, such as O_2_•^−^, H_2_O_2_, and •OH, whereas type II reactions primarily generate singlet oxygen (^1^O_2_) [26]. Although both pathways can occur simultaneously, type II reactions predominate for most clinically used PS, making ^1^O_2_ the principal cytotoxic species [27]. Nevertheless, the relative contribution of type I and type II reactions can be modulated by photosensitiser design, local oxygen concentration, and light-delivery parameters [28]. For example, recent iridium-based PS illustrate how photosensitiser design can shift this photochemical balance. Liu et al. developed an iridium complex (Ir-1) designed to favour type I •OH generation over the more oxygen-dependent type II production of ^1^O_2_, thereby retaining photodynamic activity under hypoxic conditions relevant to the tumour microenvironment [29]. Similarly, the iridium(III) complex Ir-pbt-Bpa was reported to generate both ^1^O_2_ and O_2_•^−^, illustrating that type I and type II photochemistry can also coexist within a single PS [30].

PS accumulate in different organelles depending on their physicochemical properties [31]. Lipophilic PS typically associate with the membranes of the endoplasmic reticulum (ER), Golgi apparatus, and mitochondria, whereas more hydrophilic compounds preferentially localise to lysosomes [31]. Consequently, the subcellular localisation of PS largely determines where RONS are generated and, therefore, which cellular structures are exposed to oxidative damage [32].

### 2.3. Non-Thermal Plasma Therapy

NTP is a partially ionised gas generated at near-ambient temperature by applying a strong electric field to a carrier gas [8,33]. The resulting plasma initiates the formation of a complex mixture of RONS, which is further shaped by interactions with ambient oxygen, nitrogen, and water vapour and continues to evolve when the plasma makes contact with liquids or biological tissue (Figure 1, right) [34,35,36]. Consequently, plasma exposure can generate a chemically diverse environment containing both ROS and reactive nitrogen species (RNS), including, among others, •OH, ^1^O_2_, O_2_•^−^, nitric oxide (NO•), peroxynitrite (ONOO^−^), H_2_O_2_, nitrite (NO_2_^−^), nitrate (NO_3_^−^), and ozone (O_3_) [8,36,37,38,39].

The relative contribution of individual RONS can vary substantially with the plasma source and operating conditions, including the carrier gas, discharge configuration, power, and treatment duration [36]. For example, Arndt et al. showed that different gas carrier modes generated distinct reactive chemistries: an oxygen-dominated mode favoured H_2_O_2_ formation and oxidative tryptophan modifications, whereas a nitrogen-dominated mode promoted NO_2_^−^ and NO_3_^−^ formation and nitrative modifications, resulting in different cellular responses [40]. Similarly, Lin et al. showed that, in a microsecond-pulsed DBD system, increasing the pulse frequency from 50 to 500 Hz over a fixed 10 s treatment increased the concentrations of several short- and longer-lived RONS, including NO•, O/O_3_-derived species, ONOO^−^, H_2_O_2_, NO_2_^−^, and NO_3_^−^ [8].

Importantly, the species initially formed within or near the plasma are not necessarily those that ultimately interact with biological targets. Highly reactive species are generally restricted to the immediate reaction environment, whereas more persistent species can accumulate in the liquid phase and contribute to biological effects beyond the initial plasma exposure [34,35,41,42]. This distinguishes direct plasma exposure, in which cells or tissue are present during plasma application, from treatment with a previously plasma-exposed liquid, where the biological response is mediated by species that persist after plasma generation. The RONS composition experienced by cells, therefore, reflects the evolution of this reaction network rather than simply the composition generated at the point of discharge.

## 3. From RONS to Anti-Tumour Immunity

### 3.1. RONS as Immunological Signals

In the context of anti-tumour immunity, RONS differ not only in their capacity to induce oxidative damage, but also in the biological targets they are able to reach and modify. Chemical reactivity, lifetime, diffusion capacity, and molecular selectivity collectively shape this behaviour (Table 1). A highly reactive species may, therefore, exert substantial oxidative effects while remaining confined to its immediate molecular environment, whereas a less reactive and more persistent species can influence targets located further from its site of formation [43,44,45].

The importance of this relationship is illustrated by •OH and ^1^O_2_. Their short lifetimes and restricted diffusion mean that their biological effects depend strongly on where they are generated [48,62]. •OH formed near cellular membranes can abstract hydrogen from polyunsaturated fatty acids and initiate lipid peroxidation, whereas formation close to nuclear DNA results in oxidative base modifications and strand breaks [78,79]. Such DNA damage can subsequently contribute to the accumulation of cytosolic DNA fragments and activation of nucleic-acid-sensing pathways [80]. Similarly, ^1^O_2_ reacts preferentially with unsaturated lipids and cholesterol and with susceptible amino acid residues, including cysteine, methionine, tryptophan, tyrosine, and histidine [81]. Its restricted range, therefore, couples its site of generation closely to the cellular structures that undergo oxidative modification.

Other RONS can influence biological processes beyond their initial site of formation. H_2_O_2_ is comparatively persistent and can cross biological membranes through aquaporins, allowing it to reversibly oxidise redox-sensitive cysteine residues and participate in intracellular redox signalling [55]. It can also perturb ER redox homeostasis, thereby promoting protein misfolding and activation of the unfolded protein response (UPR) [57,58]. Thus, differences in RONS persistence do not simply alter the extent of oxidative damage but also determine whether oxidative effects remain spatially restricted or can be transmitted to other cellular compartments.

Reactive nitrogen chemistry adds another layer to this spatial and temporal behaviour. NO_2_^−^ and NO_3_^−^ are comparatively stable and can serve as reservoirs from which more reactive nitrogen species are generated under appropriate biological conditions [73,74]. In hypoxic and acidic environments, NO_2_^−^ can contribute to NO• formation [82]. NO• can modify susceptible protein targets and react with O_2_•^−^ to form ONOO^−^, extending the spectrum of oxidative and nitrosative modifications to lipids, proteins, and DNA [69,70,83]. Consequently, the biological response to RONS reflects not only the properties of the species initially generated, but also their localisation, accessible molecular targets, and subsequent chemical transformations [84].

### 3.2. From Oxidative Stress to ICD

Because different RONS preferentially target specific subcellular compartments, they do not activate every component of the ICD programme to the same extent [85]. Oxidative injury to the endoplasmic reticulum, mitochondria, and nuclear or mitochondrial DNA can each contribute to the emission of ICD-associated danger signals, although their relative importance varies with the RONS profile, treatment conditions, and cellular antioxidant capacity [7,86].

The ER is particularly sensitive to oxidative disturbance because protein folding and calcium homeostasis require a tightly controlled redox environment [87,88]. RONS-mediated disruption of these processes can increase the burden of misfolded proteins and activate ER stress signalling [88]. In ICD-relevant settings, phosphorylation of eIF2α, often involving the PERK arm of the integrated stress response, has been linked to the relocalisation of calreticulin (CRT) from the ER lumen to the cell surface [88]. Surface-exposed CRT can facilitate recognition and engulfment of stressed or dying tumour cells by antigen-presenting cells, thereby contributing to the efficient acquisition of tumour antigens [89]. The extent to which other UPR branches, including IRE1 and ATF6, participate depends on the cellular context [88].

Oxidative stress can also generate immunologically relevant DNA damage [90]. Incomplete repair of damaged nuclear DNA, or release of mitochondrial DNA following mitochondrial injury, can increase the availability of double-stranded DNA in the cytosol [91]. Where cytosolic DNA accumulates, cGAS-dependent activation of STING can induce type I interferon-associated signalling [92]. This pathway may enhance antigen-presenting-cell activation and support the priming of tumour-reactive T cells, although the magnitude and functional importance of this response differ between tumour models and treatments [2].

Mitochondria provide a further link between oxidative injury, metabolic dysfunction, and inflammatory signalling [11]. Damage to respiratory-chain components, mitochondrial membranes, or mitochondrial DNA can impair oxidative phosphorylation and increase mitochondrial RONS production [93]. This may amplify ER stress and favour the generation or cytosolic release of mitochondrial DNA, thereby linking mitochondrial dysfunction to both calreticulin exposure and DNA-sensing pathways [93].

ICD-associated DAMPs are emitted at different stages of the cellular stress and death response. ATP can be released while plasma-membrane integrity is still maintained and can provide chemotactic and activating cues for myeloid cells [94]. By contrast, HMGB1 becomes extracellular predominantly during later stages of cell death as membrane integrity is lost, where it can influence antigen processing and innate immune sensing [2]. Thus, RONS-induced ICD should be understood as a coordinated but context-dependent stress response in which calreticulin exposure, ATP secretion, HMGB1 release, and type I interferon-associated signalling collectively shape the final immunogenic response [12]. Consequently, differences in the RONS profiles reported for RT, PDT, and NTP would be expected to influence the relative engagement of these pathways, providing the mechanistic basis for the modality-associated immunogenic patterns.

### 3.3. Immune Consequences of RONS-Induced ICD

RONS-induced ICD can initiate anti-tumour immunity by converting stressed or dying tumour cells into a source of antigens and immunostimulatory signals. ER stress can promote the pre-apoptotic surface exposure of CRT, which facilitates phagocytic uptake of dying tumour cells by antigen-presenting cells [89]. Extracellular ATP acts as a chemoattractant for myeloid cells and activates purinergic signalling in dendritic cells, whereas HMGB1 promotes antigen processing and presentation through pattern-recognition receptor signalling [95,96]. Together, these signals can promote dendritic-cell recruitment, uptake of dying tumour cells, and maturation [86].

Conventional type 1 dendritic cells (cDC1) are particularly important in this process because of their capacity to cross-present tumour antigens on MHC class I [97]. Following antigen acquisition in the tumour, cDC1 can migrate to tumour-draining lymph nodes and prime tumour-specific CD8^+^ T cells [98]. Effective RONS-induced anti-tumour immunity, therefore, requires more than DAMP emission, as it also depends on sufficient cDC1 recruitment, activation, and cross-presentation capacity [99].

**Figure 2:**
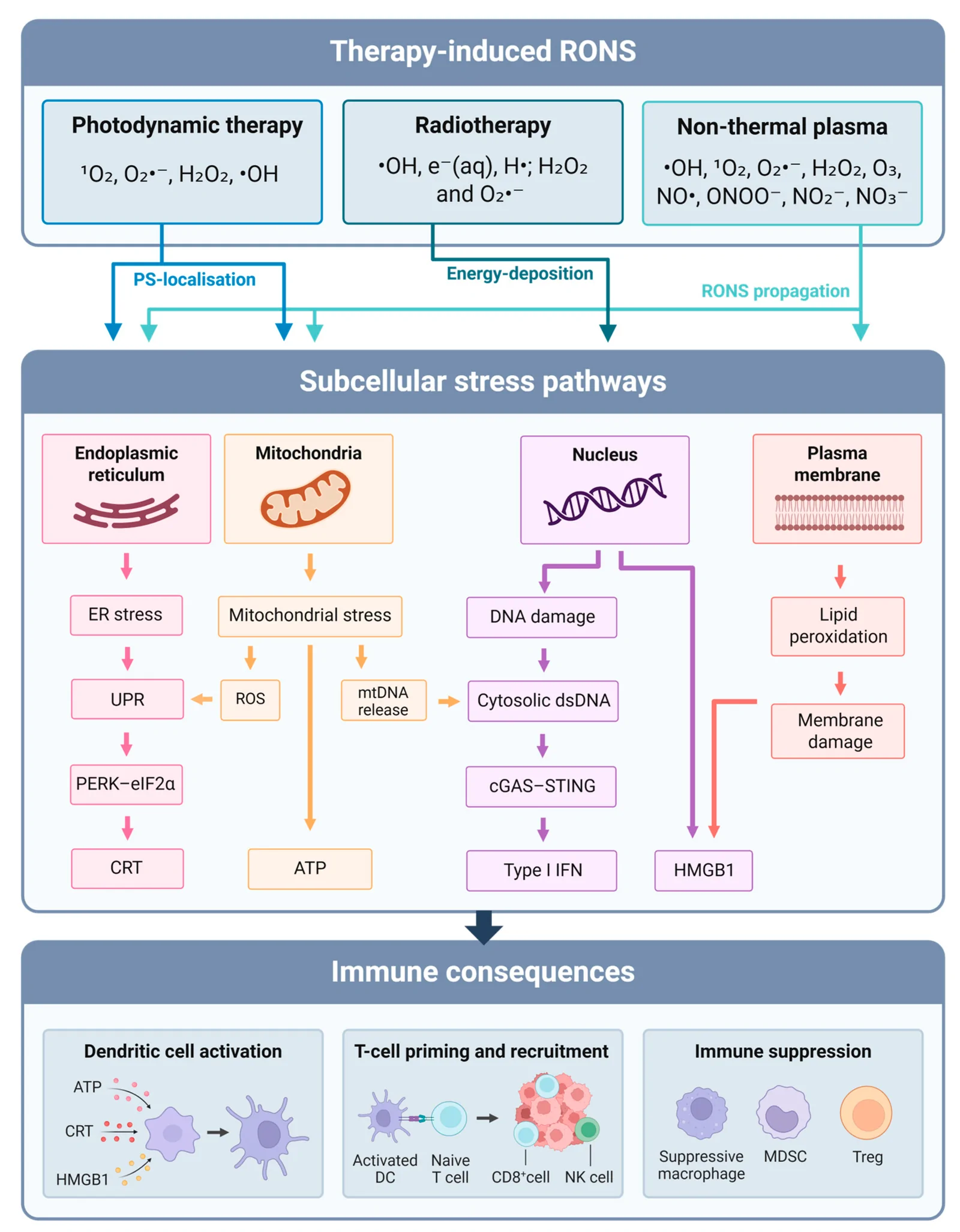
Framework linking therapy-induced reactive oxygen and nitrogen species (RONS) to subcellular stress pathways and immune consequences. Radiotherapy (RT), photodynamic therapy (PDT), and non-thermal plasma therapy (NTP) generate distinct but overlapping RONS profiles. Their composition and spatial distribution influence the relative engagement of subcellular stress pathways. For PDT, the initial site of oxidative stress is determined mainly by photosensitiser (PS) localisation, commonly involving the endoplasmic reticulum (ER) or mitochondria. RT deposits ionising energy throughout the irradiated cell, although nuclear DNA damage represents the predominant pathway illustrated here. NTP-derived RONS are delivered from the extracellular environment and can affect the plasma membrane, ER, and mitochondria through RONS and oxidative stress propagation. ER stress and the unfolded protein response (UPR) can promote PERK–eIF2α-dependent calreticulin (CRT) exposure. Mitochondrial dysfunction can contribute to reactive oxygen species (ROS) amplification, ATP secretion, and mitochondrial DNA (mtDNA) release, while damaged nuclear or mitochondrial DNA can generate cytosolic double-stranded DNA (dsDNA) and activate cGAS–STING-dependent type I interferon (IFN) signalling. Plasma-membrane lipid peroxidation and loss of membrane integrity can further contribute to high-mobility group box 1 (HMGB1) release. Together, these signals can support dendritic-cell activation, T-cell priming, and the recruitment or activation of CD8^+^ T cells and natural killer (NK) cells. Their translation into productive anti-tumour immunity is influenced by suppressive macrophages, myeloid-derived suppressor cells (MDSCs), and regulatory T cells (Tregs) within the tumour microenvironment. The indicated routes represent predominant or commonly reported, context-dependent patterns rather than fixed or modality-exclusive mechanisms. Created in BioRender. Privat Maldonado, A. (2026) https://BioRender.com/e41zzik (accessed on 28 July 2026).

DNA damage and mitochondrial DNA release can provide an additional amplification pathway. Cytosolic DNA activates cGAS–STING signalling and promotes type I interferon production, which further supports dendritic-cell maturation and CD8^+^ T-cell priming [100]. Type I interferon-associated signalling can also induce the CXCR3 ligands CXCL9 and CXCL10 in cDC1 and other myeloid cells, supporting the recruitment of effector CD8^+^ T cells and NK cells into the tumour [97]. NK cells may reinforce cDC1 recruitment through CCL5 and XCL1/XCL2, while oxidative stress can alter tumour-cell expression of NK-cell ligands [101]. Whether these processes produce a durable immune response depends on the local melanoma microenvironment. Macrophages can support clearance of dying cells and inflammatory signalling, but tumour-associated macrophage states may instead favour immunosuppression and tissue remodelling [102]. Similarly, myeloid-derived suppressor cells and regulatory T cells constrain T-cell activation through metabolic competition, suppressive mediators, and inhibition of co-stimulatory signalling [103]. RONS-induced ICD may, therefore, support the development of a T-cell-inflamed tumour microenvironment, but it does not independently guarantee productive anti-tumour immunity [2]. Figure 2 summarises the proposed framework linking therapy-associated RONS to compartment-specific stress pathways, ICD-associated signals, and downstream immune consequences.

## 4. How RONS Characteristics Shape Immunogenic Outcomes

Direct comparisons between RT, PDT, and NTP remain limited, as the three modalities have rarely been evaluated in the same melanoma model using matched treatment schedules, equivalent tumour burdens, and a common set of immunological endpoints. The literature, therefore, supports context-dependent tendencies rather than definitive modality-intrinsic differences. The comparison in this review is organised in three sections: where RONS are generated and where oxidative damage begins (Section 4.1), which intracellular stress pathways and DAMP profiles have been documented (Section 4.2), and how far these signals have been shown to translate into immune activation (Section 4.3). Table 2 provides a non-exhaustive comparison between RT, PDT, and NTP across these stages of RONS-mediated ICD.

### 4.1. Site of RONS Generation and Initial Oxidative Damage

A principal difference between the three modalities lies in how RONS are generated, since this determines both where oxidative stress develops and which reactive species predominate.

In PDT, oxidative damage is expected to remain close to the organelle in which the photosensitiser (PS) accumulates. The principal effector under type II photochemistry, ^1^O_2_, has an intracellular lifetime below approximately 3 µs. Its corresponding diffusion distance ranges from tens to a few hundred nanometres and is, therefore, shorter than the diameter of most organelles (Table 1) [6,81]. Damage is, therefore, concentrated in the membranes and proteins immediately surrounding the site of generation [104]. Because PS localisation depends on physicochemical properties, this compartment varies between agents: lipophilic PSs concentrate at ER, Golgi, or mitochondrial membranes, whereas more hydrophilic compounds accumulate preferentially in lysosomes [6].

Melanoma studies illustrate this variability. Lamberti et al. observed preferential ER localisation of protoporphyrin IX generated from methyl-aminolevulinate (Me-ALA) in B16-OVA cells, identifying this organelle as the early site of oxidative injury in their model [105]. In other settings, the initial photodynamic injury is directed towards mitochondria. Wang et al. demonstrated mitochondrial accumulation of the iridium(III) complex Ir-pbt-Bpa in A375 cells, with the structurally related ER-localising complex Ir-Bpa producing no detectable CRT translocation under comparable conditions [30]. This pair of compounds provides one of the clearest indications that the compartment of oxidative damage, rather than PS chemistry alone, shapes the downstream response. Nuclear localisation is less common but mechanistically relevant: PS-conjugated modular nanotransporters carrying a nuclear-localisation sequence achieved intranuclear accumulation of bacteriochlorin p in Cloudman S91 and B16-F1 tumours in vivo [106]. This approach remains preclinical, however, and differs from clinically used PSs, which localise predominantly to the ER or mitochondria [107,108].

In contrast to PDT, RT does not depend on accumulation of an exogenous agent in a specific organelle but deposits ionising energy throughout the irradiated cell [109]. Several compartments may, therefore, be affected, although nuclear DNA remains the principal target [109]. Warters et al. followed the induction and rejoining of radiation-induced double-strand breaks through nuclear γ-H2AX foci in different human melanoma cell lines and found detection and rejoining kinetics comparable to those of normal melanocytes [110]. The same study reported markedly elevated basal foci in melanoma cells (approximately 7–17 per nucleus, compared with 0.4 in melanocytes) that colocalised with ATM, BRCA1, 53BP1, Nbs1, and TRF1, so that foci returned to this raised baseline rather than to zero after irradiation [110]. The contribution of repair capacity was further illustrated by Noh et al., who showed that cyclic AMP signalling modulated the DNA-damage response and non-homologous end joining through protein kinase A-dependent phosphorylation of DNA-PKcs [111]. However, the direction of this effect varied across the tested cell types, which included SK-MEL-2 and SK-MEL-28 melanoma cells [111].

Mitochondrial and ER responses modify the processing of this primary nuclear injury rather than acting as independent sites of initial damage. Ren et al. showed that ionising radiation induced mitochondrial structural damage and Parkin/BNIP3-dependent mitophagy in murine B16 and S91 melanoma, and that this mitophagy enhanced rather than limited DNA damage [112]. Meng et al. found that SIRT7 depletion increased irradiation-induced apoptosis in cutaneous melanoma cells together with ER-stress signalling, including eIF2α acetylation and phosphorylation and induction of DDIT3, XBP1, and GRP78 [113]. These findings indicate that mitochondrial quality control and ER-stress adaptation influence how melanoma cells respond to irradiation, without identifying either compartment as a predominant site of initial damage.

NTP differs from both PDT and RT in that its RONS are generated predominantly outside the cell, at the plasma–liquid or plasma–tissue interface [41]. The plasma membrane, therefore, forms the first cellular compartment encountered, making membrane lipid peroxidation one of the earliest effects [114]. In B16 melanoma cells, NTP increased malondialdehyde, a lipid peroxidation product [115]. This increase was of similar magnitude in non-malignant L929 fibroblasts, whereas the NO• response was stronger in the melanoma line [115]. Direct evidence of membrane lipid oxidation after NTP exposure in melanoma remains limited, however. Studies in head-and-neck and other epithelial cell lines using the lipid-peroxidation-sensitive probe BODIPY 581/591 C11 nevertheless support its occurrence [116]. In these models, lipid oxidation was greater immediately after NTP treatment [116].

Following the initial oxidative effects at the cell surface, NTP-derived stress can propagate to the ER, mitochondria, and nucleus. In Mel Im and Mel Juso cells, Schneider et al. found that the NTP-induced rise in cytosolic calcium originated largely from intracellular stores [117]. Pharmacological inhibition indicated contributions from ryanodine-receptor-mediated release from the ER and from mitochondria [117]. Zimmermann et al. further demonstrated activation of the unfolded protein response in the same two cell lines [118]. NTP-activated solutions have also been associated with mitochondrial dysfunction, increased intracellular oxidative stress, DNA damage, and direct exposure increases nuclear γ-H2AX in A375 melanoma cells [119]. The available melanoma literature, therefore, supports a progression from membrane-proximal RONS delivery towards interconnected ER, mitochondrial, and nuclear stress responses, which distinguishes the spatial pattern of NTP from the PS-directed oxidative stress of PDT and the predominantly nuclear damage of RT.

### 4.2. Intracellular Stress Pathways and DAMP Profiles

At the mechanistic level, all three modalities are capable of engaging the principal stress pathways associated with ICD: ER stress and the unfolded protein response, cytosolic DNA sensing through cGAS–STING, and mitochondrial dysfunction. None appears restricted to a single pathway. What differs between studies is the relative contribution of these pathways under the specific conditions examined, and the DAMP profiles reported for each modality reflect these conditions rather than a fixed modality signature.

For PDT, ER stress is most consistently associated with CRT exposure, while ATP secretion and HMGB1 release accompany the subsequent death response. In B16F10 cells, Morais et al. showed that PDT with an aluminium-phthalocyanine nano-emulsion induced CRT exposure, ATP secretion, and HMGB1 release across a range of PS concentrations, but only cells treated at intermediate cytotoxicity (IC_50_ and IC_70_) protected mice against a subsequent live-tumour challenge [120]. Importantly, increasing cytotoxicity did not translate into greater functional immunogenicity, though DAMP exposure was maintained. Konda et al. observed the same three DAMPs after PDT with the structurally similar ruthenium(II) photosensitisers ML19B01 and ML19B02, yet with different resulting DAMP profiles, indicating that closely related compounds can differ in the immunogenic signals they generate. Mitochondria-targeting PDT also promote a similar DAMP profile release [121]. Irradiation of Ir-pbt-Bpa in A375 cells increased extracellular ATP and HMGB1 as well as CRT translocation [30].

The response to PDT is not limited to these DAMPs. In B16-OVA cells, Me-ALA/PpIX-PDT increased IFN-α/β transcripts within hours, with IRF3 phosphorylation, upregulation of cGAS, and induction of the interferon-stimulated genes *Cxcl10*, *Mx1* and *Isg15* [105]. STING and TBK1 were not measured in that study, however, so the interferon response is best described as cGAS-associated rather than as a fully mapped cGAS–STING axis. Thus, the available evidence does not support restricting PDT to ER stress or to a single DAMP profile. ER-centred oxidative stress damage provides an important route to CRT exposure, while the death response and, in selected models, interferon-associated signalling broaden the range of immunogenic signals. Direct comparison between studies remains difficult, however, because melanoma models, photosensitisers, localisation patterns, light fluences, sampling times, and DAMP assays all differ.

For RT, cytosolic DNA sensing and type I interferon signalling are among the most consistently documented immunogenic responses. Jagodinsky et al. evaluated external-beam irradiation in B78-D14 and B16 melanoma models using single doses of 2.5 or 12 Gy, or three daily fractions of 8 Gy [122]. They observed a delayed tumour-cell type I interferon response, with *Ifnb1*, *Mx1*, *Oas2*, and *Oas3* expression peaking approximately seven days after irradiation. This induction of type I interferon-associated genes was absent in STING-deficient (*Tmem173*^−/−^) B16 cells, supporting a requirement for tumour-cell-intrinsic STING signalling. This delayed response should be interpreted in the context of evidence from non-melanoma models showing that high single-fraction doses can induce TREX1. Importantly, Vanpouille-Box et al. showed that, in carcinoma models, high single-fraction radiation doses induced TREX1 expression, promoting cytosolic DNA degradation and attenuating cGAS–STING-dependent type I interferon signalling [123]. The onset of TREX1 induction occurred between approximately 12 and 18 Gy across the tested cell lines, but this range should not be interpreted as a universal threshold or a melanoma-specific therapeutic window. Whether a comparable TREX1 induction threshold operates in melanoma remains unresolved.

RT can also induce CRT exposure, ATP release, and HMGB1 release, although this evidence is less extensive than that for DNA damage and interferon signalling, and much of it derives from high-LET irradiation [124,125,126]. Zhou et al. evaluated the immunogenic effects of a single 5 GyE dose of carbon ions in B16 and S91 melanoma cells and human U2OS cells [124]. Irradiation induced CRT translocation, eIF2α phosphorylation, extracellular ATP release, nuclear HMGB1 loss, and a type I interferon response. Additionally, serum ATP and HMGB1 levels were also increased in tumour-bearing mice. Together, these studies support a hierarchy rather than a fixed RT-induced DAMP profile. Nuclear DNA damage and the subsequent STING-dependent interferon response are the most consistently demonstrated route, while CRT, ATP, and HMGB1 provide additional routes whose magnitude depends on radiation quality, dose, and sampling time.

For NTP, the relationship between RONS composition, intracellular stress, and DAMP release is less consistently mapped than for PDT or RT. However, several melanoma studies provide direct evidence of CRT exposure and ATP release. Using B16F10 and A375 melanoma cells, Lin et al. showed that direct DBD plasma treatment induced energy-dependent surface calreticulin exposure on viable cells [8]. Their complementary chemical and vaccination experiments indicated that short-lived plasma-derived RONS, rather than the tested persistent RONS alone, were required for the ICD-associated response. Azzariti et al. found that H_2_O_2_-enriched plasma-activated medium increased surface CRT and extracellular ATP in *BRAF*-mutant Hmel1 metastatic melanoma cells, more strongly than in HBL cells, whereas nitrite-rich medium was less effective [127]. Bekeschus et al. reported increased surface CRT, together with MHC class I and melanocortin receptor 1, in murine metastatic melanoma cells after plasma-derived oxidant exposure [128]. Among the established DAMPs, CRT exposure is, therefore, the most reproducibly reported after NTP treatment of melanoma cells, followed by ATP release, for which the evidence is more limited. HMGB1 release and cGAS–STING/type I interferon signalling remain insufficiently characterised in melanoma. Although NTP is frequently described as inducing the complete CRT-ATP-HMGB1 profile, these signals have not been quantified together in comparable melanoma models, and the interferon arm in particular remains an evidence gap rather than an established feature.

### 4.3. Translation into Immune Activation

PDT-induced DAMP emission can translate into DC uptake and maturation and, in selected models, into systemic T-cell activation. Konda et al. showed that B16F10 cells dying after ML19B01- or ML19B02-PDT were phagocytosed efficiently by bone-marrow-derived DCs, with greater uptake of ML19B01-treated cells, and that vaccination with these dying cells delayed tumour growth and improved tumour-free survival [121]. Using a solid lipid nanoparticle formulation of aluminium phthalocyanine chloride, Simões et al. found that PDT of B16F10 cells promoted DC activation [129]. DCs exposed to the treated tumour cells displayed increased MHC-II, CD80, and CD86 expression, together with increased IL-12 and IFN-γ production. These studies connect the CRT-ATP-HMGB1 profile to functional antigen capture and antigen-presenting-cell maturation. An interferon-associated component can also be recruited, however. In B16-OVA cells, Me-ALA/PpIX-PDT increased tumour-cell type I interferon signalling and, in co-culture, increased DC chemotaxis with higher CD80 and MHC-II expression [105].

Evidence for systemic effects of PDT is strongest for organelle-targeted and nanoparticle-enabled formulations. In a bilateral B16F10 model, irradiation of one tumour after administration of the mitochondria-localised complex Ir-pbt-Bpa reduced growth of both the treated and the untreated lesion, with increased CD8^+^ T-cell responses, reduced regulatory T-cell (Treg) abundance, and expansion of effector-memory T cells [30]. PDT with an aluminium-phthalocyanine nano-emulsion similarly reduced growth of an untreated contralateral tumour under one of two treatment protocols and altered peripheral CD4^+^ and CD8^+^ T-cell populations [130]. Taken together, PDT can proceed from local DAMP emission to DC activation and systemic adaptive immunity, but this outcome is established mainly for specialised delivery platforms and specific treatment protocols and cannot be assumed for PDT regimens in general.

RT can also translate nuclear DNA damage into DC activation and CD8^+^ T-cell priming, although the melanoma response depends on dose, radiation quality, and local immune context. In a B16gp model, local irradiation with 10 Gy activated tumour-associated DCs and generated tumour-specific effector CD8^+^ T cells [131]. Tumour control depended on DCs and CD8^+^ T cells, whereas CD4^+^ T cells and macrophages were dispensable. Irradiation can also improve DC antigen handling without extensive tumour-cell killing. In the poorly immunogenic D5 melanoma, irradiated tumour cells enhanced DC antigen uptake, migration to tumour-draining lymph nodes, and antigen-presentation capacity, using 60 Gy in vitro and 8.5 Gy in five fractions in vivo [132]. However, these schedules are considerably higher than those used in most clinical melanoma regimens, which limits direct extrapolation to clinical melanoma radiotherapy. Evidence from a different radiation-delivery approach further supports an increased intratumoural presence of cytotoxic T cells following irradiation. Using B16-F10-Luc tumours, Jarosz-Biej et al. found that a single 10 Gy dose of high-dose-rate contact brachytherapy markedly inhibited tumour growth and increased the intratumoural area occupied by cytotoxic CD8^+^ lymphocytes [133]. Collectively, these preclinical studies suggest that RT can promote DC-dependent antigen presentation and cytotoxic T-cell recruitment in melanoma. However, the limited number of studies and considerable variation in tumour models, radiation modalities, doses, and immune interventions prevent the identification of an optimal immunogenic regimen.

NTP can affect several immune compartments in melanoma, although these responses have not been evaluated together in a single matched in vivo model. Using complementary tumour treatment and vaccination experiments, Bekeschus et al. investigated both local immune changes and the development of systemic anti-tumour immunity [134]. Direct kINPen treatment of B16F10 tumours increased intratumoural DC and CD8^+^ T-cell abundance under the feed-gas conditions that achieved the strongest tumour control, whereas an argon/oxygen admixture reduced DC numbers. In a separate vaccination experiment in this study, splenocytes from plasma-vaccinated animals released more CXCL1, CXCL10, IFN-γ, IL-1α, IL-6, and TNF-α and less GM-CSF and CCL17 following re-exposure to melanoma cells. Moreover, half of the mice vaccinated with plasma-treated melanoma cells resisted subsequent tumour challenge, providing functional evidence of tumour-specific immunity. Tumour-derived soluble mediators may additionally contribute to the immune response following NTP treatment. Plasma-treated B16F10 cells released ATP and CXCL1 within 6 h, followed at 24 h by increased TNF-α, IL-1β, and CCL4 together with IL-10, and conditioned medium subsequently altered cytokine production and increased CD28 expression on CD8^+^ T cells in splenocyte co-cultures [135].

NTP also influences macrophage and NK-cell responses. In co-cultures containing A375 or MV3 melanoma cells, plasma-activated liquid promoted M1-like polarisation of macrophages and enhanced their suppression of melanoma-cell proliferation, with inhibitor experiments implicating ROS/JAK2/STAT1 signalling [136]. Treatment of A375 cells with the kINPen jet altered the expression of activating and inhibitory NK-cell ligands and increased IL-6 and IL-8 secretion [137]. These changes were accompanied by greater susceptibility to human NK-cell-mediated killing, while effects in non-malignant HaCaT keratinocytes were weaker or absent. The same study reported increased MICA/B and HLA class I together with increased HLA-E and PD-L1 after extended treatment, so the ligand shift is not uniformly favourable to NK-cell recognition [137]. These findings indicate that NTP can support melanoma immune control through DAMP and cytokine release, DC and CD8^+^ T-cell accumulation, macrophage polarisation, and increased NK-cell susceptibility, with the relative contribution depending on plasma chemistry, delivery mode, and cellular context.

Collectively, the available evidence indicates that the immunogenic effects of RONS-based therapies depend on more than the overall level of oxidative stress. The identity and site of generation of the reactive species, the dose and duration of exposure, and the antioxidant and metabolic state of tumour and surrounding cells influence the relative involvement of ER stress, mitochondrial dysfunction, and DNA-damage sensing. These processes, in turn, shape the resulting cell-death pathways and immune signals. RT, PDT, and NTP show predominant mechanistic tendencies under specific experimental conditions, but these pathways are neither exclusive to one modality nor fixed. Each modality can engage overlapping cellular stress and immune pathways, with their relative contribution determined by the treatment conditions and biological context.

**Table 2:** Comparative characteristics of radiotherapy (RT), photodynamic therapy (PDT), and non-thermal plasma (NTP) as inducers of reactive oxygen and nitrogen species (RONS)-mediated immunogenic cell death (ICD). Reported pathways and ICD characteristics are not mutually exclusive. The distinction between predominantly and other reported effects reflects recurring patterns across the available literature rather than a quantitatively established hierarchy, as direct comparisons under standardised experimental conditions are currently lacking.

	RT	PDT	NTP
**RONS source**	Intracellular water radiolysis [19,22]	Photosensitiser activation by light [6,25]	Extracellular, plasma-derived RONS [8,33]
**Dominant RONS**	•OH, e^−^(aq), H•; secondary H_2_O_2_ and O_2_•^−^ [19,22]	Predominantly ^1^O_2_ (type II), also O_2_•^−^, H_2_O_2_, •OH (type I) [26,27]	Interface-localised: •OH, ^1^O_2_, O_2_•^−^; diffusible: H_2_O_2_, O_3_, NO•, ONOO^−^; stable reservoirs: NO_2_^−^, NO_3_^−^ [41,138]
**Spatial RONS delivery**	Intracellular energy deposition [19]	Restricted to PS-bearing organelles [26,31]	Extracellular → propagation into cells [8,36]
**Main intracellular targets**	Nucleus, cytoplasm [23,79,80]	ER, mitochondria (depending on PS localisation) [63,139]	Cell membrane, mitochondria, ER [8,140,141]
**Most consistently reported stress pathways**	DNA damage, cGAS-STING [14,90,91]	ER stress, UPR [25,139]	Mitochondrial stress, ER stress, UPR, lipid peroxidation [8,141,142]
**Most reported ICD-associated signals**	Type I IFN, HMGB1 [14,86]	Strong CRT exposure [139]	CRT, ATP [8,15]
**Key tuning parameters**	Dose, dose rate, fractionation [123,143]	Photosensitiser localisation, drug-to-light interval, light delivery [26,104]	Carrier gas, applied voltage, treatment duration, direct vs. indirect delivery [36,144]
**Dependence on oxygen**	High [11,19]	High (especially type II) [25,27,62,63,145]	Lower, due to RNS contribution [36]
**Spatial constraint**	Not limited [146,147]	Limited by light [104,148]	Limited by RONS diffusion [33,36]
**Main advantages**	Deep penetration [147,149,150]	High spatial precision [25,148]	Adjustable RONS composition [134]
**Main limitations**	Dose window, normal-tissue toxicity, hypoxia [147,149,150,151,152]	Melanin light scatter, hypoxia, light penetration [104,153]	Limited penetration [33,134]
**Current literature with ICI**	Clinical evidence, including prospective trials; clinical benefit remains heterogeneous and a consistent survival advantage is not established [147,149,150,154,155,156]	Predominantly preclinical evidence; limited melanoma-specific combination evidence [157,158]	Exclusively preclinical evidence; limited to cell-based and syngeneic mouse models [134,159,160]

## 5. Combination of RONS-Inducing Therapies with ICIs for Melanoma

These findings indicate that RONS-based therapies can initiate several components of an anti-tumour immune response, but do not establish that they can overcome the immunosuppressive melanoma microenvironment. Checkpoint-mediated inhibition and suppression by Tregs, myeloid-derived suppressor cells, and macrophages can still restrict the persistence and effector function of tumour-reactive T cells. RONS-induced ICD can, therefore, promote tumour-antigen presentation and immune-cell activation, but is unlikely to sustain an effective T-cell response by itself. Combining RT, PDT, or NTP with ICIs is, therefore, intended to sustain this response and improve anti-tumour control. Although the three modalities engage overlapping immunogenic pathways, differences in their RONS characteristics may influence how they interact with ICIs. Each modality also has a distinct balance of immunological strengths, practical advantages, and biological or clinical limitations that should be considered when optimising combination treatment in melanoma [161].

### 5.1. RT and ICIs

RT offers the most direct mechanistic complementarity with ICIs as, unlike PDT and NTP, RT is not constrained by penetration depth and can be applied to deep-seated as well as superficial disease [162,163]. Its immunogenic effects nevertheless depend strongly on the dose delivered per fraction. Above a certain dose threshold, the exonuclease TREX1 degrades cytosolic double-stranded DNA and thereby attenuates type I interferon signalling [123]. This threshold is not a fixed value, however, but varied between 12 and 18 Gy across the cell lines tested, with 12 Gy in TSA mammary carcinoma, 15 Gy in MCA38 and 4T1, and 18 Gy in MDA-MB-231. As the models used in this study were mammary-, colon-, and lung-derived, and no melanoma line was included, the numerical window should be treated as an illustration of a mechanistic principle rather than as a melanoma-specific limit. The same study from Vanpouille-Box et al. further showed that three fractions of 8 Gy did not induce TREX1, despite a cumulative dose of 24 Gy, confirming the importance of the dose delivered per fraction rather than the total dose [123]. Consistent with this finding, only the fractionated schedule produced abscopal responses when combined with anti-CTLA-4, while TREX1 knockdown restored the immunogenicity of a single 20 Gy dose. These immunological considerations must also be balanced against normal-tissue exposure. Larger irradiated volumes increase the integral dose to surrounding tissues and can increase acute and late toxicity, thereby constraining treatment field size and the feasibility of repeated irradiation [151,152]. This limitation is particularly relevant when RT is used to treat multiple lesions or when local treatment is repeated during ongoing systemic therapy [164].

Clinically, RT is used in melanoma across distinct settings that differ in therapeutic intent, treatment technique, and the scope for interaction with systemic immunotherapy. These include lesion-directed treatment of oligoprogressive disease, symptom-directed irradiation of bone or other extracranial metastases, and stereotactic management of brain metastases. The available RT–ICI literature should, therefore, be interpreted according to the irradiated site, dose and fractionation, disease burden, and the timing of RT relative to ICIs, rather than as evidence for a uniform combination effect across clinical scenarios [165]. In the randomised phase II CHEERS trial, which included a melanoma subgroup, up to three lesions achieved 75% local control of irradiated lesions but did not improve the primary progression-free survival endpoint compared with ICI alone [156]. Brain metastases illustrate the same point from the dose side, since a single fraction controlled above 90% in melanoma requires approximately 23.4 Gy, whereas 18 Gy sufficed for small lesions [166,167]. A retrospective single-institution series of 101 patients, 70 of whom received concurrent ipilimumab and RT, reported a median overall survival of 19 months with the combination compared with 10 months with ipilimumab alone [155]. Complete response was also more frequent in the combination group (25.7% versus 6.5%). However, progression-free survival and overall response rate did not differ significantly. Furthermore, a systematic review of 16 studies and 451 patients reported a median abscopal response rate of 26.5% and a median overall survival of 19 months, but only 5 of the 16 studies were prospective and all used ipilimumab [150]. Collectively, these data support the feasibility of RT–ICI combinations but do not establish a consistent survival advantage in melanoma.

### 5.2. PDT and ICIs

PDT offers a distinct mechanistic rationale for combination with ICIs [168,169]. By restricting oxidative damage to PS-bearing organelles, it preferentially induces ER stress and CRT exposure, thereby promoting efficient recognition and phagocytosis of dying tumour cells [139]. This highly localised mechanism, together with the spatial selectivity afforded by light activation, limits damage to surrounding tissue and minimises systemic toxicity, making PDT an attractive option for repeated treatment of accessible superficial lesions [170]. PDT can also modify the target of ICIs directly. In colorectal models, PDT-generated ROS produced local hypoxia and HIF-1α stabilisation, which increased PD-L1 transcription through a hypoxia-response element in the *CD274* promoter, an effect reversed by HIF-1α inhibition or knockdown [157]. This provides a mechanistic argument for pairing PDT with PD-L1 inhibition, although the mechanism was established in colorectal and not in melanoma cells. Despite these advantages, several factors currently limit the application of PDT in melanoma. Melanin absorbs and scatters the PS, activating light while also quenching ^1^O_2_, reducing the effective oxidative dose in pigmented lesions and limiting treatment efficacy despite optimisation of irradiation parameters [153]. In addition, the oxygen dependence of type II photochemistry reduces efficacy under hypoxic conditions, while limited light penetration restricts PDT to superficial targets [148].

The available evidence for combining PDT with ICIs remains preclinical, and melanoma-specific studies constitute only a small part of this literature. In bilateral breast cancer in vivo models, nanoparticle-mediated PDT combined with anti-PD-L1 controlled both irradiated and non-irradiated tumours, consistent with a systemic tumour-specific cytotoxic T-cell response [171]. In melanoma-relevant systems, available studies indicate that PDT can alter checkpoint-related and immune-regulatory pathways, but evidence for a PDT–ICI-mediated systemic response remains incomplete. In the pheophorbide A study, PDT increased PD-L1 expression in B16-F10 melanoma cells in vitro [172]. In vivo, the authors evaluated a cRGD-modified liposomal formulation co-delivering pheophorbide A and anti-PD-L1 in 4T1 murine mammary tumours, where it improved local tumour control and was associated with DC maturation, enhanced cytotoxic T-cell infiltration, and reduced Treg abundance. A separate B16-F10 study combined an anti-PD-1 antibody with a nanomicelle platform that inhibited local tumour growth and increased immune-cell infiltration [173]. However, irradiation raised intratumoural temperature from 32 to 60.7 °C, indicating a substantial photothermal contribution. Taken together, PDT combined with ICIs is mechanistically attractive in melanoma but has not yet been shown to control non-treated lesions in pigmented melanoma models or been established clinically.

### 5.3. NTP and ICIs

Finally, NTP offers a distinct approach to combination with ICIs, as the composition of the delivered RONS can be modulated through plasma-source design and operating conditions [174]. By adjusting these, the relative contribution of immunogenic effects, including CRT exposure, ATP release, and HMGB1 secretion, can be modulated. Its limited toxicity to surrounding healthy tissue and local application make NTP particularly attractive for repeated treatment of superficial lesions [175]. In addition, because NTP-derived RONS are generated extracellularly from ambient oxygen and nitrogen, their initial composition is largely independent of intratumour oxygen availability. Once delivered to the tumour, however, hypoxic and acidic melanoma microenvironments can still modulate downstream redox signalling, for example, by favouring NO_2_^−^ to NO• conversion [159]. Despite these potential advantages, several challenges currently limit the translation of NTP to the clinic. Its immunogenic effects nevertheless remain highly dependent on treatment parameters and are generally greater following direct RONS delivery [176,177]. Moreover, the limited propagation of NTP-derived species from an extracellular source restricts clinical application to superficial and readily accessible lesions, such as those in melanoma [33]. A further challenge is the substantial heterogeneity in plasma devices and operating conditions, which can generate markedly different RONS compositions and biological effects and thereby complicate treatment standardisation and dose definition. Its immunogenic and therapeutic effects have also only been studied in preclinical models, and their translation to patients has yet to be established. Evidence for NTP–ICI combinations remains confined to preclinical models. In a murine melanoma study, combining NTP with anti-PD-1 increased tumour remission relative to either monotherapy and was associated with greater intratumoural DC and macrophage abundance [160]. The reported effect on contralateral tumours, however, was exploratory, and intratumoural CD4^+^ and CD8^+^ T-cell frequencies were not significantly increased. In a second melanoma study, NTP combined with anti-PD-L1 delivered through a hollow microneedle patch produced the strongest primary-tumour control, prolonged survival, and regression of untreated contralateral tumours [178]. These findings provide a rationale for further investigation, but do not yet establish clinical efficacy, an optimal treatment schedule, or the patient populations most likely to benefit.

## 6. Limitations

Several limitations should be considered when interpreting this review. The first is methodological heterogeneity. The available studies use different melanoma models, including pigmented and amelanotic cell lines and transfected variants with improved intrinsic immunogenicity. Treatment conditions also differ, including radiation quality and dose rate, photosensitiser localisation, and plasma-delivery format. In addition, studies use different methods to measure RONS and assess outcomes at time points ranging from minutes to days. The criteria used to identify ICD are similarly variable. Some studies measure a single DAMP, while others assess dendritic-cell activation, protection against tumour rechallenge, or control of untreated tumours. The differences observed between modalities may, therefore, partly arise from differences in experimental design. Consequently, the pathways that appear most prominent for each modality should be considered general tendencies in the available literature rather than a fixed mechanistic hierarchy.

Second, the three treatment modalities differ substantially in their stage of clinical development. RT has an established role in melanoma treatment and has been evaluated in combination with ICIs in prospective clinical trials. Evidence for PDT combined with ICIs in melanoma remains preclinical, whereas studies of NTP combined with ICIs are largely limited to cell-based experiments and syngeneic mouse models. The parallel structure of this comparison is, therefore, intended to compare the underlying mechanisms, rather than to imply that the three modalities have reached the same level of clinical validation.

Finally, the role of melanoma pigmentation remains insufficiently understood for all three treatment modalities. Melanin can reduce the effective photodynamic dose reaching pigmented lesions through light absorption and scattering and may also quench ^1^O_2_ [153]. Additionally, melanin and its oxidation products are redox-active and may also modify oxidative damage induced by RT and NTP in a context-dependent manner [179,180,181]. However, these effects have not been systematically examined in matched pigmented and amelanotic melanoma models. Furthermore, melanoma phenotype plasticity, antioxidant and mitochondrial metabolic programmes, tumour oxygenation, and lesion-specific immune context may influence both sensitivity to oxidative local treatment and the generation of systemic anti-tumour immunity [182,183,184]. Pigmentation and these interrelated melanoma-specific features should, therefore, be considered potential sources of response variability in future translational studies.

## 7. Future Perspectives

The mechanistic framework presented in this review suggests that optimisation of oxidative therapies should extend beyond local tumour control to include immunogenicity as a treatment objective. Although RT, PDT, and NTP ultimately converge on overlapping pathways associated with ICD, their distinct RONS characteristics influence the relative contribution of ER stress, DNA-damage sensing, and mitochondrial dysfunction. A better understanding of how treatment parameters, including RT dose and fractionation, PS localisation and illumination conditions, and plasma source and operating settings, shape these immune-relevant pathways will be essential for defining modality-specific immunogenic windows and for the rational integration of oxidative therapies with ICI.

Comparative studies using matched tumour models, standardised RONS characterisation, and common molecular and functional ICD endpoints would provide critical validation of the mechanistic framework proposed here. These studies should determine whether modality-associated differences persist when treatment conditions are controlled and should assess not only DAMP emission but also dendritic-cell activation, tumour-specific T-cell responses, control of untreated lesions, and survival.

Translation of these approaches, particularly their combination with ICIs, will require simultaneous optimisation of efficacy and safety. RONS-mediated tumour stress must be sufficient to initiate ICD and local immune activation without causing unacceptable injury to normal tissue or exacerbating local and systemic immune-related toxicity. Future studies should, therefore, define modality-specific therapeutic windows that account for treatment dose and sequence, local tissue effects, and systemic immune-related adverse events.

Ultimately, clinical translation remains the central challenge. RT–ICI combinations already benefit from a comparatively mature clinical evidence base, whereas PDT–ICI and particularly NTP–ICI combinations remain supported predominantly by preclinical studies. Expanding well-designed translational and early-phase clinical studies will, therefore, be essential to determine whether the mechanistic advantages identified for PDT and NTP translate into meaningful clinical benefit. Finally, integrating mechanistic understanding with clinical validation will determine how RT, PDT, and NTP can be most effectively incorporated into personalised immunotherapeutic strategies for melanoma.

## 8. Conclusions

RT, PDT, and NTP can each induce RONS-mediated cellular stress and ICD-associated signalling in melanoma, but they should not be considered interchangeable inducers of anti-tumour immunity. Differences in RONS composition, site of generation, lifetime, and diffusion shape the relative involvement of ER stress, mitochondrial dysfunction, DNA damage, and cytosolic nucleic acid sensing. These pathways can promote CRT exposure, ATP secretion, HMGB1 release, and type I interferon signalling, thereby supporting dendritic-cell activation and tumour-specific T-cell priming.

The available evidence nevertheless identifies modality-associated tendencies rather than fixed or exclusive immunogenic profiles. RT is commonly associated with nuclear DNA damage and type I interferon-related responses, whereas the effects of PDT depend strongly on photosensitiser localisation and frequently involve oxidative injury to the ER and subsequent CRT exposure. NTP can engage membrane, ER, mitochondrial, and nuclear stress pathways, although their relative contribution depends strongly on treatment conditions. Across all three modalities, these responses are further influenced by tumour oxygenation, pigmentation, and the redox and immune context of the lesion. The detection of individual DAMPs should, therefore, not be regarded as evidence of functional ICD or durable systemic anti-tumour immunity.

RONS-induced ICD may provide antigenic and adjuvant signals that complement ICIs, but it has not been established that these signals alone can overcome checkpoint-mediated inhibition or the immunosuppressive melanoma microenvironment. RT currently has the most developed clinical evidence base in combination with ICIs, although the optimal immunogenic treatment schedule and its capacity to provide a consistent survival benefit remain unresolved. Evidence for PDT–ICI and NTP–ICI combinations remains predominantly preclinical. Studies using matched melanoma models, standardised RONS characterisation, and shared molecular and functional immune endpoints are, therefore, needed to determine how these modalities can be integrated safely and rationally with ICIs.

## Figures and Tables

**Table 1:** Physicochemical parameters and predominant biological targets of reactive oxygen and nitrogen species (RONS).

RONS	Half-Life ^1^	Diffusion Distance ^1^	Membrane Permeability	Primary Reactivity	Main Biological Targets	Ref.
•OH	~10^−9^ s	~6 nm	No	Strong/non-selective	DNA backbone and bases, membrane lipids, most amino acids, carbohydrates	[46,47,48,49]
e^−^_(aq)_	~10^−7^ s	~1 nm	No	Very strong, moderately selective	Nucleobases, electron-deficient molecules, and metal centres	[48,50,51]
H•	~10^−6^ s	~1 nm	No	Strong, moderately selective	Unsaturated lipids, thiols, proteins, and nucleic acid constituents	[50,52]
H_2_O_2_	~1 s	~1 µm	Yes	Weak, selective	Peroxiredoxins, glutathione peroxidases, catalase, phosphatases, kinases, and redox-sensitive transcriptional regulators	[53,54,55,56,57,58]
O_2_•^−^	~10^−6^ s	~30 nm	No	Weak, selective	Iron–sulphur clusters, mitochondrial enzymes, metalloproteins	[59,60,61]
^1^O_2_	~10^−6^ s	~100 nm	Yes	Strong, selective	Unsaturated lipids, cholesterol, histidine, methionine, tryptophan, cysteine, and guanine	[27,62,63,64]
NO•	~10^−2^ s	~500 µm	Yes	Weak, selective	Soluble guanylate cyclase, haem proteins, iron–sulphur clusters, thiols	[60,65,66,67]
ONOO^−^	~1 s	~4 µm	Yes	Strong, moderately selective	Thiols, metal centres, tyrosine residues, DNA, membrane lipids, and mitochondrial proteins	[61,68,69,70]
NO_2_^−^	~1 h	≫1 µm	No	Very weak (reservoir)	Haemoglobin and myoglobin (reductive NO• formation), proteins and lipids under low pH, extracellular fluids	[71,72,73,74]
NO_3_^−^	~4 h	≫1 µm	No	Very weak (reservoir)	Blood and tissue fluids, transport systems	[71,72,74]
O_3_	~10^−4^ s	>1 µm	No	Strong, selective	Unsaturated membrane lipids, cholesterol, thiols, methionine, tryptophan, lipid hydroperoxides	[75,76,77]

^1^ Reported half-lives and diffusion distances represent order-of-magnitude estimates under near-physiological aqueous and cellular conditions. These parameters can differ between experimental systems and specific microenvironments.

## Data Availability

No new data were created or analysed in this study. Data sharing is not applicable to this article.
